# Commissioning of Mobius3D/FX for patient‐specific quality assurance: The CUIMC—NewYork Presbyterian Hospital experience

**DOI:** 10.1002/acm2.14183

**Published:** 2023-10-17

**Authors:** Fiona Li, Michael Price

**Affiliations:** ^1^ Department of Radiation Oncology Columbia University Irving Medical Center New York New York USA

**Keywords:** Action limit, MLC Log file‐based QA, Mobius3D/FX, Patient‐specific quality assurance, Tolerance limit

## Abstract

**Purpose:**

To present the process undertaken by our institute in commissioning Mobius3D (M3D) for patient‐specific quality assurance.

**Method:**

168 plans were randomly selected to compare dose distribution measured with ArcCheck and dose calculated from M3D, both compared against the treatment planning system (TPS). The gamma criteria for measurement and M3D are 3%/2 mm with 10% and 50% dose thresholds, respectively. The effect of tissue inhomogeneity was investigated on 11 plans by recalculating the dose in M3D on a homogeneous phantom. Tolerance and action limits were established following the AAPM Task Group 218 recommendations.

**Results:**

The M3D showed high variability in gamma passing rate compared to the measurement. Twenty‐three out of 168 plans had false negative dose comparisons. These plans fall under high tissue inhomogeneity like lung and metal implants, small field targets, and breast plans planned with high energy. One false negative case (0.6%) was observed. A single tolerance limit of 91% and 92% gamma passing rate for the M3D and measurement‐based PSQA were established, respectively. Against the expectation, recalculating plans on the homogeneous phantom in M3D did not necessarily increase the gamma passing rate. These plans have a duty cycle >4.2, and the small field sizes combined with differences in slice thickness contributed to observed dose differences in the homogeneous phantom comparisons.

**Conclusion:**

Following the commissioning, M3D is adopted in our institute. Currently, the gamma criteria used for measurement and M3D are 3%/2 mm, 40% dose threshold, with gamma passing rates of 92% and 95%, respectively. A higher passing rate for M3D is adopted until more data is available. The combined effect of plan modulation, the field sizes, the tissue inhomogeneity, the dose algorithm, and the volume averaging effect from differences in slice thickness can contribute to the differences in dose in M3D and TPS.

## 1 INTRODUCTION

With the advancement of technology in radiation oncology and better interpretation of the radiobiological effect of tissues, there is a demand for more concise and accurate treatment delivery. Within the past decade, the number of treatments delivered using static intensity modulated radiation therapy (IMRT) and volumetric modulated arc therapy (VMAT) has significantly increased. Unlike 3D plan delivery, IMRT and VMAT are delivered with static and rotational gantry delivery, respectively, with fluence defined by complex multi‐leaf collimator (MLC) motion.

IMRT and VMAT are inverse planning processes to create a homogeneous high‐target dose distribution while sparing the organs at risk (OARs). However, due to the restrictive dose constraints on the OARs and the inherent properties of the beam geometry, steep dose gradients are created around the OARs. The result is a conformal non‐uniform target dose distribution. The dose is optimized by dividing the beams into beamlets of varying intensity, translating it into a set of MLC instructions for beam delivery. The constant motion of the MLCs with varying dose rates creates variable fluence maps, making it difficult to measure using scanning chamber.^1^ The complex treatment delivery demands that individual plans undergo patient‐specific quality assurance (PSQA) before delivery. The process of PSQA is analogous to an end‐to‐end test that is expected to spot errors like data transmission errors, mechanical errors at the machine, and TPS planning dosimetric mistakes during the treatment delivery.^2^ Even a tiny uncertainty of 1 mm in the MLC position can create errors in dose by a few percent.^3^ Hence the precise location of the MLC leaf is necessary for accurate dose measurement.

Clinics utilize several different techniques of PSQA. One of the first methods is using films or ionization chambers for measuring point dose. Films are tedious and time‐consuming, and measuring the point dose may not appropriately represent the patient plan. 2D and 3D doses can be measured using diode or ionization chamber arrays (e.g., SunNuclear MapCheck) arranged in planar or cylindrical arrays (e.g., SunNuclear ArcCheck). This requires creating a verification plan in the treatment planning system (TPS) to mirror the actual setup of the dosimeter phantom for measurement. The plan is created using the same parameters as the treatment plan. The other commonly used method is electronic portal image dosimetry (EPID) which does not require phantom setup. While EPID has the advantage of high spatial resolution, the detectors are not tissue equivalent, are not sensitive to gantry errors, and have size limitations.^2^, ^4^

Another method that is gaining traction in PSQA is MLC log file‐based QA. The MLC log file depends on the ability of the machine to record the position of the MLC leaves accurately as the gantry rotates around the patient, the MU delivered for each MLC segment, and the collimator and gantry rotations. Different measurement devices are sensitive to other kinds of errors, with most having poor sensitivity to identifying unacceptable plans.^2^, ^4^, ^5^, ^6^ Mobius3D/FX (M3D/MFX; Varian Medical System, Palo Alto, CA, USA) is an excellent alternative to PSQA with results similar to the measurement based PSQA,^7^, ^8^, ^9^ which is sensitive to small collimator and MLC bank errors.^10^, ^11^ M3D is a commercially available secondary check software that performs an independent collapsed cone convolution superposition algorithm (CCCS) for comparison with the planned dose calculated from TPS.^12^ The MFX component automatically retrieves MLC log files recorded from the delivered treatment plan in the machine. It recalculates the dose distribution on patient CT data using the data retrieved from the delivery log files. The dose distribution is compared using the commonly used metric, the gamma analysis.

Currently, our institute utilizes SunNuclear ArcCheck (SunNuclear Corporation, Melbourne, FL) for dose measurements. ArcCheck requires phantom setup and is sensitive to setup errors, especially for small field plans. Since it is a tedious process, it can only be performed after clinic hours or during relatively long gaps in patient treatments. Conversely, M3D does not require phantom setup, eliminating setup errors, and can be delivered during regular clinic hours. The dose is calculated on patient CT images, which considers tissue inhomogeneity. It is a closer representation of the “true” dose distribution. In addition, the regions where the gamma criteria fail are indicated in M3D. The software is already utilized for secondary MU checks in our clinic. This paper presents the transition from measurement‐based PSQA to log‐file‐based PSQA using M3D/FX and the procedure for commissioning M3D/FX for PSQA.

## 2 METHOD

### 2.1 Plan selection

A total of 168 VMAT and IMRT plans, from January—June of 2022, were randomly selected for the commissioning of M3D/FX for PSQA. At our center, all the static IMRT plans are delivered via a Varian Trilogy linear accelerator, and rotational VMAT plans via Varian TrueBeam (TB) linear accelerators. Plans were selected without considering treatment type, site, or nominal beam energy.

### 2.2 Dose comparison between measurements and Mobius3D

All the plans were calculated with the Acuros XB algorithm (V 16.0.3) in Eclipse TPS V15.6 (Varian Medical System, Palo Alto, CA, USA). Phantom measurements were performed with an ArcCheck and compared, filed‐by‐field, to the dose determined by the TPS for an equivalent phantom setup using commercial software (SNC Patient ver 6.7.4). The gamma criteria used for measurement was 3%/2 mm and 10% dose threshold with global normalization.

For M3D/FX log‐file‐based PSQA, the treatment plans need to be delivered by the linear accelerator to generate the MLC log files for analysis. The presence or absence of a patient or phantom in the beam's path does not affect file generation. For dose comparison, the dose was calculated on patient CT images using the 3D gamma criteria of 3%/2 mm and 50% dose threshold with global normalization. M3D was set to calculate the dose with automatic grid size detection. The grid size is independent of the TPS and is calculated based on the smallest effective field size within a given plan. Occasionally, when the dose grid in TPS is small (∼1.25 × 1.25 × 1.25 cm^3^), especially for small field SBRTs, the M3D dose grid is adjusted to larger grid sizes due to memory limits. The mismatch in the dose grid voxels may affect the gamma passing rate.

### 2.3 Tolerance and action limit

AAPM task group 218^13^ recommendations were used to establish the tolerance and action limits. According to the task group, the “tolerance limit” is a limit within which the process usually operates. Beyond the limit, the process might be changing, but it is at the discretion of the responsible physicist to determine if action is required. The “action limit” is observed as a limit that, once broached, may harm the patient, so intervention is required.^13^, ^14^ For simplicity, the process to which these concepts are applied is the overall PSQA process.

Due to varying modulations in the plans, they were divided into stereotactic body radiation therapy (SBRT) and non‐SBRT groups. A universal limit, applicable to all institutes, or a site‐specific limit based on clinical data or clinical experience can be established. The action limit from the task groups utilizes process action limit formula^14^, ^15^:

$$\Delta A\;=\;\beta \sqrt{\sigma^{2}+{\left(\bar{x}-T\right)}^{2},}$$

where ΔA is the difference between the upper and lower action limits, typically written as $\pm \frac{A}{2}$. *T* is the process γ passing rate ( = 100%), and σ^2^ and $\bar{x}$ are the process variance and mean, respectively. β considers the desired level of quality required and is suggested to be “6” for PSQA to include six standard deviations of process variability. σ^2^ and $\bar{x}$ are calculated after the process is brought into control by resolving the underlying reason behind the undesirable behavior.

The tolerance limit is determined using an I‐chart, a statistical method to measure the variability in the system and identify out‐of‐control processes or data outliers. It consists of process mean, which is the mean of individual gamma passing rates, and the control limits are the upper and lower limits defined by equations below^14^, ^15^: The limit defines the tolerance limits.

$$\mathrm{Center}\;\mathrm{line}\;=\;\frac{\sum_{1}^{n}x}{n}$$

$$\mathrm{Control}\;\mathrm{limits}\;=\;\mathrm{center}\mathrm{line}\;\pm \;2.66.\;\bar{\mathrm{mR}}$$

where *n* is the number of measurements, *x* is the individual measurement, and $\bar{mR}=\frac{\sum_{i=2}^{n}\left|x_{i}-x_{i-1}\right|}{n-1}\;$ is moving range.

### 2.4 Effect of tissue inhomogeneity

Eleven plans were retrospectively chosen to determine the effect of tissue inhomogeneity in the M3D dose calculation by calculating the dose on a homogeneous phantom. Plans were randomly selected to include M3D gamma passing rates ranging from 90%−100% and one lung plan with a gamma passing rate of 79%. These plans were recalculated on ArcCheck phantom, utilized for clinical plan verification, as a close representation of homogeneous phantom. M3D's 3D dose from the homogeneous phantom was compared to the corresponding plans calculated on the patient CT images.

## 3 RESULTS

### 3.1 Comparison of measurement and Mobius3D

**FIGURE 1:**
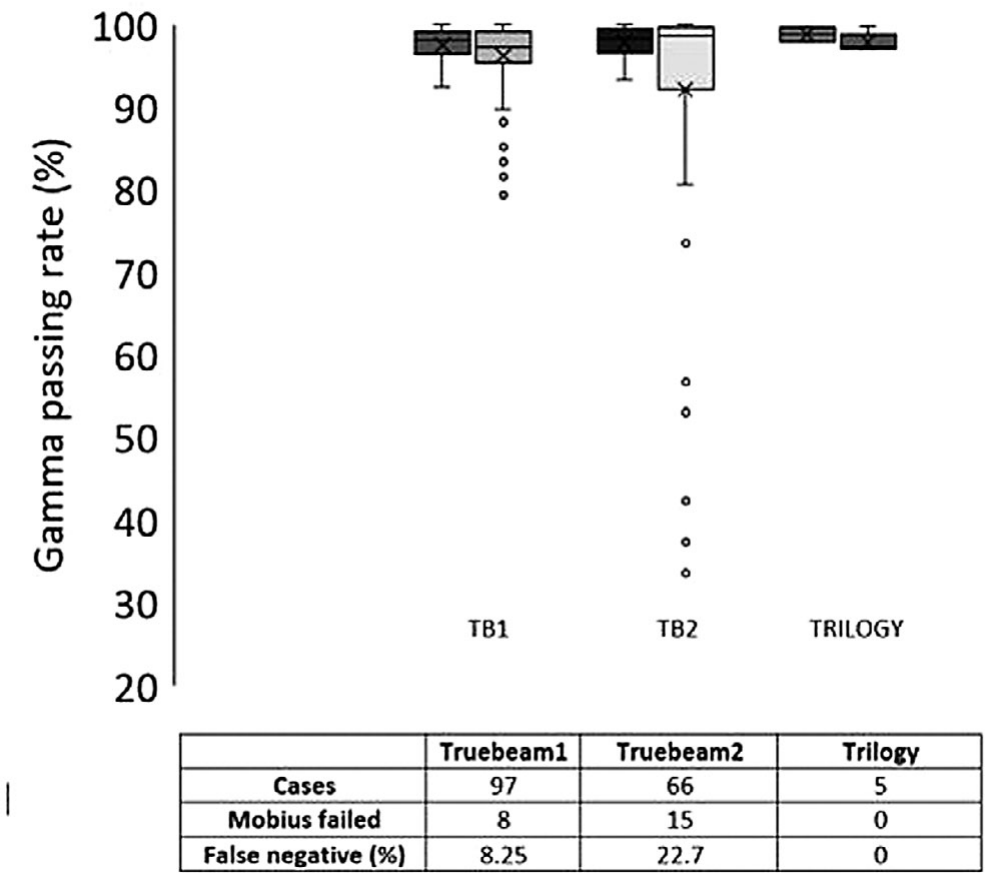
Patient cases used for plan comparison between the ArcCheck measurements and MobiusFX dose calculations per linear accelerator. The left and right bars under each machine are representative of measurement and MFX gamma passing rates, respectively. The result indicates a large variation in the MFX gamma passing rates.

As shown in Figure 1, the 168 plans are divided into three machines. Static IMRT plans were mainly treated at the Varian Trilogy linear accelerator, while VMAT plans by the TrueBeam platform. Most of the plans were treated with VMAT, so only 5 Trilogy‐delivered plans were compared. Of the 168 plans, 23 failed MFX, but the measurement‐based PSQA passed using the 90% gamma passing rate. Only one plan failed measurement but passed the MFX gamma criteria. Using measurement‐based PSQA as a reference, the false negative rate is 13.7%, and the false positive rate is 0.6% for MFX.

**FIGURE 2:**
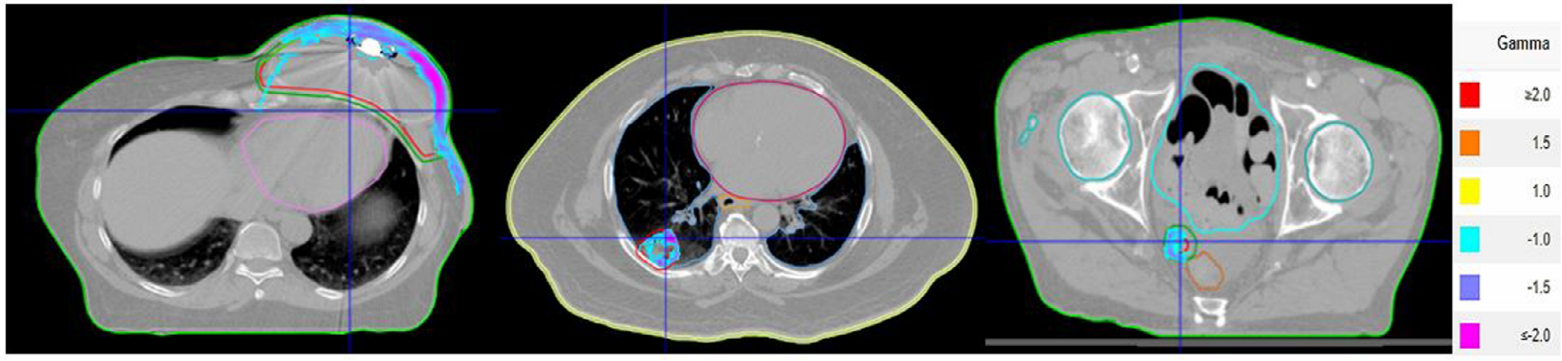
False negative Mobius3D plans fall under four categories: Breast plans with high energy (Left), lung plans with tissue inhomogeneity (Middle), small field plans (Right), and appendages. The color wash region indicates a region outside the gamma value of −1.0 and 1.0 with the gamma value scale shown on the right.

The plans under the false negative bracket consist of lung plans, SBRT plans containing small fields (< 4 × 4 × 4 cm^3^), appendages (near the bone or metal implants), and breast plans that were planned with a high energy beam (10 MV) (Figure 2). The large dose differences observed in the breast plans are in the build‐up and supraclavicular lymph node regions. The one false positive case is a head and neck plan.

### 3.2 Tolerance and action level

According to the AAPM task group 218 and Taweap et al.,^13^, ^14^ the PSQA process must be stable or controlled. If the process is not controlled, then the reason behind the out‐of‐control process needs to be eliminated. From the plan comparisons, MFX failed due to dose inhomogeneity in the lung and the dose uncertainty in the buildup region, as in the breast plans and small field plans. Since the reasons behind the out‐of‐control process cannot be eliminated, plans that failed the MFX gamma passing rate but passed measurement PSQA were removed by determining action and tolerance limits. This resulted in removing a total of 21 patients from the patient cohort.

Table 1 shows the tolerance and action level determined per machine and categorized by the treatment types (SBRT vs. non‐SBRT). As expected, the tolerance and action limit for SBRT is much lower than the non‐SBRT levels. The plans for SBRT are all planned with flattening filter‐free (FFF) beams with smaller field sizes and higher MU modulation. In addition, most of the lung plans that failed Mobius3D are also planned with FFF beams.

**TABLE 1:** Action and tolerance limits per machine and treatment types. The average values for combined machines were calculated without considering machine types.

			Tolerance		Action	
	Machine	Type	Measurement	MFX	Measurement	MFX
	Trilogy	IMRT	97	94	95	92
	TB1	SBRT	95	81	81	75
	TB1	non‐SBRT	93	91	91	90
	TB2	SBRT	85	84	84	71
	TB2	non‐SBRT	94	94	94	93
**Average**	**TB (1&2)**	**non‐SBRT**	**93.5**	**92.5**	**92.5**	**91.5**
	**TB (1&2)**	**SBRT**	**90**	**82.5**	**82.5**	**73**
	**Trilogy**	**6X**	**97**	**94**	**95**	**92**

Bold significance to seperate the main results from rest of the table

Table 2 shows the tolerance and action limit by combining all plans per machine. The limits for Truebeam‐delivered plans are very close to each other. Since only 5 plans were delivered using a Trilogy linear accelerator for comparison, the final limits were established using the average of action and tolerance limits for the Truebeam‐derived comparisons.

**TABLE 2:** Action and tolerance limits for no consideration of treatment types.

		Tolerance		Action		
	Machine	Measurement	MFX	Measurement	MFX	*N*
	**Trilogy**	97	94	95	92	5
	**TB1**	92	90	90	89	88
	**TB2**	92	92	91	91	54
**Average**	**TB**	**92**	**91**	**90.5**	**90**	**142**

Bold significance to seperate the main results from rest of the table

### 3.3 Effect of tissue inhomogeneity

Table 3 compares the M3D gamma passing rate for 11 random treatment plans that were calculated on both homogeneous phantom and patient CT images, along with the treatment sites and the max MU duty cycle as determined by Eclipse TPS. The MU duty cycle is defined as the ratio of the total MU delivered and dose per fraction. It is a measure of the plan's overall modulation. The gamma criteria used for Mobius3D is 3%/2 mm and 50% dose threshold.

**TABLE 3:** Comparison of plans calculated, in M3D interface, on both homogeneous phantom and patient CT images in terms of 3D gamma passing rate.

			3D gamma passing rate (%)		
Plan number	Site of treatment	Duty cycle $\left(=\frac{totalMU}{dose/fx}\right)$	Patient CT (a)	Homogeneous (b)	% Difference $\left(=\frac{b-a}{b}\times 100\right)$
1	Scapula^*^	2.33	90.3	91.4	+1.2
2	Scapula^*^	1.99	95.4	95.2	−0.2
3	Prostate^*^	4.31	93.6	90.8	−3.1
4	Prostate^*^	4.67	91.6	87.7	−4.4
5	Prostate	3.45	99.7	100	+0.3
6	Lung^*^	3.63	79.1	87.1	+9.2
7	Pancreas^*^	2.97	91.6	82.6	−10.4
8	Spine^*^	4.73	93.3	89.8	−3.4
9	Chest Wall	3.44	90.1	97.6	+7.7
10	Lymph node	4.13	97.6	95.7	−2.0
11	Spine^*^	4.07	97.6	98.1	+0.5

* Indicates SBRT treatment.

Against the expectation, the gamma passing rate for homogeneous phantom was not better than that calculated on patient CT for all plans (see Table 3). The plans with better performance on the patient CT (>2%) were cases where the duty cycle was greater than 4.2, except for plan #7. The average (± standard deviation) gamma index passing rate percentage differences between the patient CT and homogeneous phantom for the sample size of 11 patients is 3.85% ± 3.67%.

## 4 DISCUSSION

M3D/FX was commissioned according to the Varian Mobius3D commissioning guidelines.^16^ The comparison of measurement PSQA and M3D/FX's 3D gamma criteria was performed to transition measurement‐based PSQA to log‐file‐based PSQA in our clinic. It should be noted that the measurements using ArcCheck were done field‐by‐field instead of true composite dose comparison. Field‐by‐field comparison is more stringent as it is sensitive to slight differences in dose and distance error. Unlike the composite dose comparison, the dose errors in one field are not “smeared” or compensated by errors from other delivered fields.^14^ Since a more stringent dose comparison was practiced at our institute, comparing M3D with our current standard was appropriate. In addition, a 10% dose threshold was used for measurements, while 50 % was used for M3D. Using lower dose thresholds, dose to OARs, are also included in the dose comparison. For global normalization, the dose differences are normalized to the prescription dose, which can artificially boost the gamma passing rate in low‐dose regions. The effect is especially prominent in 3D dose distribution, where more points with low‐dose regions exist. Hence, a 50% dose threshold would provide a better dose comparison for M3D.

According to the Varian Mobius commissioning guidelines, it is expected that the gamma passing rate for complex plans should be greater than >90% using less stringent gamma criteria of 5%/3 mm.^12^ With our more stringent criteria of 3%/2 mm, the false negative (14%) was considerably high. About 25% of the plans at our clinic failed the MFX gamma passing criteria. In this case, our policy is to perform a measurement‐based PSQA to verify the plan passing rate. M3D also performs a Plan Check by recalculating dose distribution using the CCCS algorithm with information obtained from imported DICOM files. It compares the dose distribution with the TPS dose distribution and computes 3D gamma criteria. From our comparison, the gamma passing rates from MFX and Plan Check match for all cases. This will be true provided the machine has no mechanical issues or errors. Thus, Plan Check's 3D gamma passing rate could be a surrogate indicator if measurement QA is necessary. For reasons unknown, other than a suspected small target region (13 cm^3^), a plan with a boost to the head and neck failed measurement but passed MFX gamma criteria. It only accounted for 0.6% of the total cases compared.

A past study has shown that M3D dose calculation is comparable to the ArcCheck measurement if the same dose algorithm was used.^10^ For our comparison, the dose distributions were calculated using two different algorithms, Boltzmann transforms (ACUROS) and CCCS, that consider tissue inhomogeneity with different approaches. Dose distributions were also compared using a homogeneous phantom to rule out the tissue inhomogeneity effect. Against the expectation, gamma criteria for four of the eleven plans had a worse passing rate on homogeneous phantom than on patient CT. Except for one plan, these plans had high duty cycle or MU modulation > 4.2. Plan #7 is a pancreas plan with a relatively small target (28 cm^3^), and the CT images and the homogeneous phantoms were scanned at two different slice thicknesses of 1 and 2 mm, respectively. The combined effects of small field and volume averaging could be the basis for worse performance on a homogeneous phantom. This agrees with the study performed by Hillman et al. that lung inhomogeneity, sharp dose fall‐off, especially near the spinal cord, and small targets are some of the contributing factors to dose disagreement between M3D and TPS.^17^ Moreover, M3D does not adequately model the MLC tongue and groove design and there is dose uncertainty for fields smaller than 3 × 3 cm^2^ . ^[^ ^7^ ^]^ The result on phantom inhomogeneity was based on a very small sample size of 11. To establish the required 95% confidence, a larger sample size >55 will be required.

Action and tolerance limits were established by TG 218 recommendations.^13^ Since the limits depend on the machine and treatment types, it was initially compared per machine and treatment type. However, the SBRT's action and tolerance limit is considerably lower than the non‐SBRT plan (Table 1). SBRT plans are highly modulated with a high‐duty cycle as they typically contain a high dose gradient with heterogeneous structures surrounding the target region. In addition, the VMAT dosimetric leaf gap (DLG) correction factor (CF) from M3D commissioning was estimated by combining both the SBRT and non‐SBRT VMAT plans. From experience, VMAT and static IMRT plans require two separate DLG CF, and this was implemented in Ethos Mobius3D V4.0.2. This could mean that SBRT and non‐SBRT VMAT plans may also require a separate DLG CF. Only five plans were compared on the Trilogy machine, and this was not taken into consideration when setting limits. The limits were recalculated by combining all the plans to set more stringent limits. Since action and tolerance limits were close, the tolerance limit was chosen as a universal limit. The concern with using field‐by‐field for measurement was resolved by remeasuring the plan using true composite plans for 15 plans (not shown). Except for the plans that did not perform well on M3D (see Figure 1), the dose distribution between the measurements and MFX was comparable.

The paper demonstrated the process undertaken at our institute during the commissioning of M3D/FX for PSQA. Log‐file based PSQA utilizing M3D is not a new concept. Lee et al., demonstrated the feasibility of utilizing M3D for PSQA. The study found a small difference in the point dose measurement between the ion chamber and other dose calculation algorithms like AAA, CCCS, and AcurosXB. The percentage mean dose difference between M3D and EPID was only 0.46%.^18^ Similarly, Basavatia et al., compared MFX with conventional QA methods and determined that 3%/3 mm was an appropriate gamma criterion for PSQA when utilizing MFX.^8^ Jolly et al., established action and tolerance limits for M3D based on 1000 M3D results. The paper utilized statistical mean and standard deviation to establish the limits for remedial action before treatments.^19^ Conversely, our paper dives into the practical aspect of commissioning PSQA. Other than establishing the action and tolerance limits based on TG‐218, the paper delved into the conditions in which M3D dose calculation may not agree with measurement‐based QA, especially for highly modulated plans and in the presence of tissue inhomogeneity. The paper serves as a good guide for transitioning from measurement based to log‐file based PSQA.

## 5 CONCLUSION

While M3D/FX is increasingly used for PSQA, it needs to sufficiently take small field uncertainty and tongue and groove effect into consideration.^13^ Though plan modulation plays a critical role in dose calculation, no correlation between the modulation and gamma passing criteria was found. Since the American College of Radiology (ACR) does not explicitly state that PSQA can be performed with log‐file based PSQA and MFX is not widely used as PSQA, a more restrictive gamma passing rate of 95% was established in the institute's MFX PSQA policy. The gamma criteria for measurement‐based PSQA and MFX were also matched to 3%/2 mm and 40% dose thresholds. While the aim was to entirely transition PSQA to a measureless process, about 25% of PSQA are still performed by measurement. With more data collected and as the community is more confident in log‐file based PSQA, less stringent gamma criteria can be adopted.

## AUTHOR CONTRIBUTIONS

F.L. is responsible for collecting, analyzing, and authoring most of the manuscript. M.P. collectively assisted with the work's experimental design, analysis methods, and manuscript editing.

## CONFLICT OF INTEREST STATEMENT

The authors declare no conflicts of interest.
